# Backward Walking and Dual-Task Assessment Improve Identification of Gait Impairments and Fall Risk in Individuals with MS

**DOI:** 10.1155/2020/6707414

**Published:** 2020-09-08

**Authors:** Erin M. Edwards, Deborah A. Kegelmeyer, Anne D. Kloos, Manon Nitta, Danya Raza, Deborah S. Nichols-Larsen, Nora E. Fritz

**Affiliations:** ^1^Translational Neuroscience Program, Wayne State University, Detroit MI, USA; ^2^Program in Physical Therapy, Wayne State University, Detroit MI, USA; ^3^Division of Physical Therapy, The Ohio State University, Columbus, OH, USA; ^4^School of Health and Rehabilitation Sciences, The Ohio State University, Columbus, OH, USA; ^5^Department of Neurology, Wayne State University, Detroit MI, USA

## Abstract

**Background:**

Individuals with multiple sclerosis (MS) experience deficits in motor and cognitive domains, resulting in impairment in dual-task walking ability. The goal of this study was to compare performance of forward walking and backward walking in single- and dual-task conditions in persons with MS to age- and sex-matched healthy controls. We also examined relationships between forward and backward walking to cognitive function, balance, and retrospective fall reports.

**Methods:**

All measures were collected in a single session. A 2 × 2 × 2 mixed model ANOVA was used to compare differences in forward and backward walking in single- and dual-task conditions between MS and healthy controls. Spearman correlations were used to examine relationships between gait and cognitive function, falls, and balance.

**Results:**

Eighteen individuals with relapsing-remitting MS and 14 age- and sex-matched healthy controls participated. Backward walking velocity revealed significant differences between groups for both single-task (*p* = 0.015) and dual-task (*p* = 0.014) conditions. Persons with MS demonstrated significant differences between single- and dual-task forward and backward walking velocities (*p* = 0.023; *p* = 0.004), whereas this difference was only apparent in the backward walking condition for healthy controls (*p* = 0.004). In persons with MS, there were significant differences in double support time between single- and dual-task conditions in both backward (*p* < 0.001) and forward (*p* = 0.001) directions. More falls at six months were significantly associated with shorter backward dual-task stride length (*r* = −0.490; *p* = 0.046) and slower velocity (*r* = −0.483; *p* = 0.050).

**Conclusion:**

Differences in MS and age- and sex-matched healthy controls are more pronounced during backward compared to forward walking under single- and dual-task conditions. Future work with a larger sample size is needed to validate the clinical utility of backward walking and dual-task assessments and mitigate the limited sensitivity of the current dual-task assessments that primarily rely upon forward walking.

## 1. Introduction

Multiple sclerosis (MS) is a progressive neurologic disease that causes debilitating motor, cognitive, and sensory impairments [1]. While a broad spectrum of clinical signs and symptoms are experienced by persons with MS, walking impairment is one of the most commonly reported symptoms to negatively impact quality of life [2–4]. Accordingly, standardized clinical measures, including the Timed 25 Foot Walk, 2-Minute Walk Test, and 6-Minute Walk Test, [5] have been established in MS to provide clinicians with informative “snapshots” of walking performance. To date, scores on clinical measures of walking performance in MS have been linked to a variety of key factors (i.e., falls, cognition, dynamic balance control) [6]. However, these measures have limited ability to detect performance fluctuations in both motor and cognitive domains that may present outside of the clinical setting, as the measures primarily rely upon single-task, forward walking measures.

Dual-task walking assessments that require individuals to perform multiple tasks simultaneously often result in a decrement in performance of one or both tasks in persons with MS [7]. Given that walking in daily life is rarely practiced without concurrent cognitive demands or secondary motor tasks, dual-task walking assessments are more generalizable to everyday life [8] and thus may improve upon the current measures of single-task forward walking. Additionally, dual-task walking requires increased cognitive and motor demands [9]. Due to the high prevalence of cognitive (65%) and motor deficits (85%) in MS [10], it is not surprising that impairments in dual-task walking are seen in individuals with MS [11–14] and have been related to cognition, fall risk [15], dynamic balance control [7], and connectivity in the supplemental motor area [16]. Moreover, dual-task performance is modulated by neurobiological systems [17, 18] affected in MS, like the dopaminergic system [19]. Collectively, these facts suggest that dual-task assessments are sensitive to key motor and cognitive processes in MS. However, it is important to note that the current dual-task walking measures utilize only forward walking [20, 21], and therefore, do not detect impairments and fall risk in persons with MS elicited during more challenging backward stepping or walking activities [22, 23]. Thus, identifying potential modifications to improve the limited sensitivity of dual-task walking measures is critical.

Recently, backward walking has been recognized as a sensitive clinical measure of mobility and fall risk in the elderly [24, 25] and other neurodegenerative disease populations [26]. Similar to dual-task walking, backward walking requires increased cognitive demands and postural control [27, 28] than forward walking. In MS, deficits in balance and postural control are increased during backward walking and significantly correlate with severity on clinical measures of forward walking and disability [29]. Additionally, when administered with a secondary cognitive task, deficits in backward walking are more prominent than during forward walking [30]. Regarding the relationship between backward walking and falls in MS, our laboratory demonstrated that backward walking velocity is a strong and unique descriptor of retrospective fallers, whereas forward walking measures were not [31]. Our laboratory also demonstrated a positive relationship between clinical measures of cognitive function and backward walking velocity [32]. Collectively, however, previous research on dual-task backward walking in MS has been limited by a small sample size and lack of demographic matching between MS and control groups [30] and has not examined the relationships of spatiotemporal gait measures to cognitive function, balance, or falls. Additionally, our laboratory's studies have primarily focused on single-task backward walking. Thus, relationships between backward walking dual-task measures and cognitive function, balance, and falls remain unidentified in MS.

Therefore, our study compared spatiotemporal gait parameters of forward walking and backward walking in single- and dual-task conditions in persons with MS and age- and sex-matched healthy controls. Additionally, we compared the relationships between forward walking and backward walking spatiotemporal measures to measures of cognitive function, retrospective fall reports, and balance. We hypothesized that persons with MS would demonstrate greater deficits in backward walking single-task and dual-task walking performance compared to forward single-task and dual-task walking performance than healthy controls. Additionally, we hypothesized that backward walking would exhibit stronger correlations to cognitive function, retrospective fall reports, and balance in comparison to forward walking in persons with MS. Identification of relationships between single- and dual-task backward walking to cognitive function, falls, and balance would aid in the development of practical, cost-efficient, and clinically feasible tools that may sensitively detect critical underlying processes that are commonly impacted by MS and related to fall risk (i.e., motor and cognitive function), thereby improving upon the current forward walking methods.

## 2. Methods

Eighteen individuals with relapsing-remitting MS were recruited from a parent study exploring training effects of video-game exercise. Fourteen age- and sex-matched healthy controls were recruited through fliers, posts on the university research database, and by word-of-mouth. Inclusion criteria for individuals with MS included 30-59 years of age, a diagnosis of relapsing-remitting MS, and an Expanded Disability Status Scale (EDSS) score between 1.0 and 5.5. Age- and sex-matched healthy controls were included if they were within 2 years of the participant with MS and of the same sex. Both MS and healthy control participants were excluded if they reported an orthopedic, neurologic, or cognitive impairment that would limit participation in study assessments. All measures were collected in a single session. The Institutional Review Board at The Ohio State University approved this study. All participants signed consent forms before participating.

### 2.1. Mobility Measures in Both MS and Controls

#### 2.1.1. GaitRite

Spatiotemporal measures of gait were acquired with the GAITRite electronic walkway (V3.9, MAP/CIR Inc.; Franklin, NJ). The GAITRite is reliable and valid for use in individuals with MS (Givon, 2009). Individuals ambulated across the GAITRite at a self-selected, comfortable pace for three trials per each of the four conditions: (1) forward, (2) forward + cognitive task (serial 3 subtraction starting at 97), (3) backward, and (4) backward + cognitive task (serial 3 subtraction starting at 95). We *a priori* chose to evaluate a limited number of gait variables, including velocity, stride length, and double support time. These variables were chosen because stride length and double support time have been linked to balance in MS [33], elderly [25], and other neurodegenerative populations [34], and backward walking velocity has specifically been linked to falls in older adults [24].

### 2.2. Additional Mobility Measures in MS

*Walking While Talking Test* (*WWTT*) requires the participant to perform walking, in which their time is recorded, under three conditions: (1) walk 40 feet with 180° turn at midpoint, (2) condition 1 + recite alphabet aloud (WWTT-Simple), and (3) condition 1 + recite alternate letters of alphabet aloud (WWTT-Complex) [35]. The WWTT is a reliable and valid test to identify older individuals at high risk for falls [35]. Additionally, poor performance on the WWTT-Complex (>33 seconds) accurately predicts elderly fallers [35].

*Timed Up and Go* (*TUG*) requires the participant to stand from a chair, walk 10 feet, turn, walk back, and sit down [36]. The TUG is reliable in MS [37]. The TUG-Cognitive (TUG-C) requires performance of the TUG with a simultaneous serial-3 subtraction task; this modification of the TUG measures dual-task performance. A time of >15 seconds to complete the TUG-Cognitive accurately predicts fallers in MS with a sensitivity of 73% [38].

*Berg Balance Scale* (*BBS*) is a 14-item measure of balance and fall risk requiring individuals to perform a variety of activity such as turning in a circle, stooping down to pick up an object, and reaching forward. Items are scored from 0 (cannot perform) to 4 (normal performance) with a maximal score of 56. The BBS is reliable in persons with MS [39–41].

*Dual-Task Questionnaire* (*DTQ*) is a 10-item subjective questionnaire of everyday tasks involving dual tasking, such as walking while talking or listening, spilling a drink while carrying it, and completing an activity while talking. Individuals are asked to rate each item for frequency of difficulty performing from 0 to 4 (0 = never; 4 = very often) [42].

#### 2.2.1. Retrospective Fall History

The number of past falls over six months was assessed by self-report. All participants were asked, “Have you fallen within the last six months? If yes, how many times?” Falls were operationally defined at the time of the retrospective fall data collection as an “unexpected event that resulted in an unintentional landing on the ground or a lower surface” [43].

### 2.3. Cognitive Measures in MS and Controls

#### 2.3.1. Symbol Digit Modalities Test (SDMT)

Participants received a key with nine numbers each corresponding to a symbol and were asked to determine the number belonging with a series of symbols using this key. The score is the number of correct answers in 90 seconds. The SDMT is a validated and reliable test in MS to analyze attention and information processing speed [44].

#### 2.3.2. Word List Generation (WLG)

The WLG is measure of verbal fluency and semantic retrieval, reliable, and validated for use in persons with MS [45]. Participants were asked to name as many animals as possible in 90 seconds.

### 2.4. Statistical Analyses

A priori power analysis based on Wajda et al. indicates sample size of 8 per group needed for 80% power to detect a change in performance between groups [30]. Descriptive statistics (mean ± standard deviation) were calculated for all variables. Outlier assessment was performed using the Shapiro-Wilk Test and box-and-whisker plots. If the data did not fit the assumptions of normality, nonparametric statistics were utilized. Mann–Whitney tests were used to compare the average ages of MS and healthy controls. Following the methods of Wajda et al., a 2 × 2 × 2 mixed model ANOVA was used to compare differences in the spatiotemporal gait parameters for forward walking, backward walking, forward dual-task walking, and backward dual-task (walking direction and dual-task condition as the within-subject factors) walking between MS and healthy controls (between subject factor) for each velocity, stride length, and double support time. Bonferroni corrections were used to examine factors influencing any observed differences. Spearman correlations were used to examine relationships between cognitive performance (SDMT and WLG) and both forward and backward walking performance in MS, relationships between retrospective fall reports and walking performance in MS, relationships between subjective dual-task performance on DTQ, and objective measures of dual-task performance (WWTT, TUG-C, forward dual-task walking, and backward dual-task walking in MS, and relationships between balance performance (BBS) and forward and backward walking performance.

## 3. Results

### 3.1. Demographics

Eighteen individuals with relapsing-remitting MS and fourteen age- and sex-matched healthy controls enrolled in this study. There was no significant difference in age (MS: 45.5(8.2); HC: 44.0 (8.8); *p* = 0.613) and sex (all female) between groups. For the MS group, EDSS scores ranged from 1.5 to 4, and the average time since diagnosis was 12.3 ± 6.7 years. Seventeen of eighteen individuals with MS were taking disease modifying therapies, and four of eighteen utilized an assistive device during testing. Healthy controls reported no falls in the past two or six months, whereas individuals with MS reported fourteen falls (4 individuals) and thirty-four falls (8 individuals), respectively.

### 3.2. Gait Parameters

**Figure 1:**
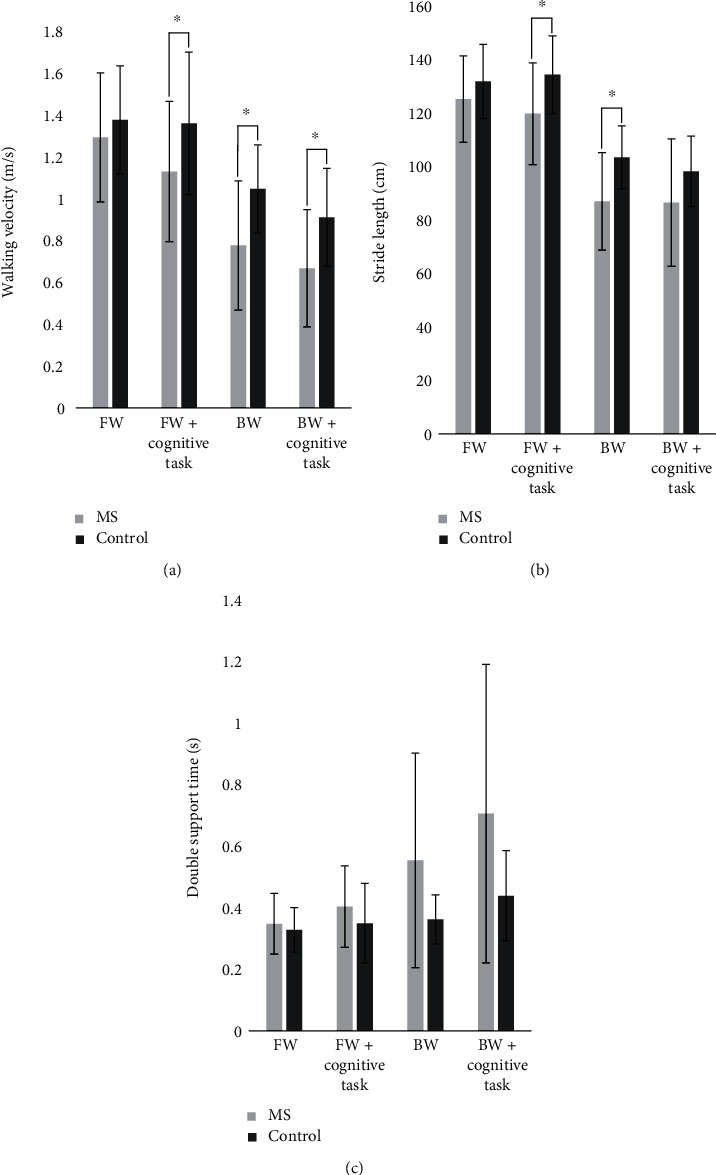
Walking velocity (a), stride length (b), and double support time (c) as a function of walking direction and group. ∗ indicates a significant difference between MS and healthy controls (*p* < 0.05).

Figure 1 demonstrates the mean and standard deviation values for velocity, stride length, and double support time for each group and walking task. Results from the ANOVA, including main effects and interactions, are outlined in Table 1, with three-way interactions depicted visually in Figure 1.

### 3.3. Velocity

There were significant differences in backward walking velocity between groups for both single- (*p* = 0.015) and dual-task (*p* = 0.014) conditions. Forward walking velocity showed no significant difference between groups for both single- (*p* = 0.087) and dual-task (*p* = 0.502) conditions. Persons with MS showed a significant difference between single- and dual-task velocity in both forward (*p* = 0.023) and backward (*p* = 0.004) directions, whereas healthy controls only showed a significant difference in the backward walking condition (*p* = 0.004; *p* = 0.827 for forward walking). No interactions were observed for walking velocity (Table 1); thus, it is likely that the differences observed between groups (*p* = 0.043) are driven by main effects of direction (*p* < 0.001) and condition (*p* = 0.005).

### 3.4. Stride Length

There was a significant difference in forward walking stride length between groups for the dual-task condition (*p* = 0.010) and backward walking stride length for the single-task condition (*p* = 0.039). In persons with MS, there was a significant difference between single- and dual-task stride lengths in both the forward (*p* < 0.001) and backward directions (*p* < 0.001). This difference was also apparent in healthy controls (*p* < 0.001) for both forward and backward walking. No interactions were observed for stride length (Table 1); thus, it is likely that the differences observed between groups (*p* = 0.046) are driven by the main effect of direction (*p* < 0.001).

### 3.5. Double Support Time

There was no difference in double support time between individuals with MS and healthy controls during single-task forward (*p* = 0.573) and backward walking (*p* = 0.285) as well as dual-task forward (*p* = 0.071) and backward walking (*p* = 0.057). In persons with MS, there were significant differences in double support time between single- and dual-task conditions in both forward (*p* = 0.001) and backward (*p* < 0.001) directions. No differences were observed in controls (*p* = 0.581; *p* = 0.295). In persons with MS, there were significant differences in double support time between forward and backward walking directions under both single- (*p* = 0.034) and dual-task (*p* < 0.001) conditions. No differences were observed in controls (*p* = 0.440 and *p* = 0.095, respectively). There was a significant interaction (*F* = 14.2; *p* = 0.001; *η*^2^ = 0.33) observed for group x direction (Table 1).

### 3.6. Relationships Among Walking, Cognitive Function, Retrospective Falls, and Balance in Persons with MS

#### 3.6.1. Cognitive Function

Better performance on the SDMT was significantly associated with longer stride length in both the forward walking condition (*r* = 0.505; *p* = 0.032) and the forward dual-task condition (*r* = 0.603; *p* = 0.008) but was not significantly associated with any other walking measures. WLG performance was not associated with any walking measures. Subjective dual-task performance on the DTQ was not significantly associated with forward or backward dual-task spatiotemporal parameters, or the WWTT, but was significantly associated with TUG-Cognitive (*r* = 0.551; *p* = 0.022) performance, with more self-reported difficulty with dual tasks associated with longer time to complete the TUG-Cognitive.

#### 3.6.2. Retrospective Falls

Falls at six months were not associated with forward walking or forward dual-task performance; however, falls at six months were significantly associated with backward dual-task velocity (*r* = −0.483; *p* = 0.050) and stride length (*r* = −0.490; *p* = 0.046) with slower velocity and shorter stride lengths associated with more fall reports.

#### 3.6.3. Balance

Better performance on the BBS was significantly associated with increased forward walking velocity (*r* = 0.548; *p* = 0.018), decreased double support time (*r* = −0.592; *p* = 0.010), and increased stride length (*r* = 0.751; *p* < 0.001) under the single-task condition. For the forward walking dual-task condition, better performance on the BBS was significantly associated with increased stride length (*r* = 0.657; *p* = 0.003). For the backward walking single-task condition, better performance on the BBS was significantly associated with increased backward walking velocity (*r* = 0.522; *p* = 0.026), decreased double support time (*r* = −0.590; *p* = 0.010), and increased stride length (*r* = 0.492; *p* = 0.038). BBS performance was not associated with backward walking parameters in the dual-task condition.

#### 3.6.4. Differences in Performance Among Those with Lower and Higher Disability Levels

**Figure 2:**
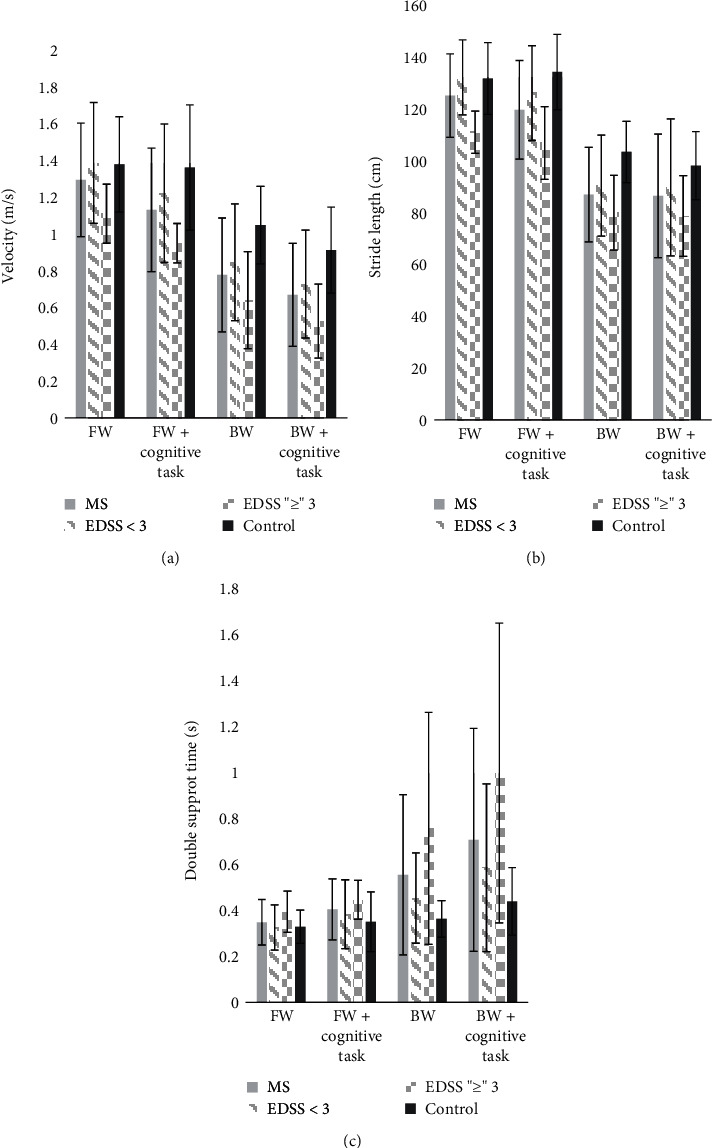
Subanalysis to examine differences in walking performance among individuals with EDSS < 3 and those with EDSS ≥ 3. MS is marked by the grey bar and HC by the black bar, with individuals with low disability (EDSS < 3) marked with diagonal lines and individuals with high disability (EDSS ≥ 3) marked with checkered blocks. Individuals with lower disability (EDSS < 3) performed similarly to healthy controls under single-task forward walking conditions but had worse performance than healthy controls in backward and dual-task conditions, particularly in backward dual-task walking.

An exploratory analysis was performed to examine differences in performance between individuals with EDSS < 3 (*n* = 12) and those with EDSS ≥ 3 (*n* = 6). Our data demonstrated that individuals with lower disability (EDSS < 3) performed similarly to healthy controls under single-task forward walking conditions but that backward walking and dual tasks, particularly in the backward walking direction, better differentiated healthy controls from those with EDSS < 3 (Figure 2).

## 4. Discussion

This study compared spatiotemporal measures in forward walking and backward walking in single- and dual-task conditions between persons with MS and healthy controls. Further, we compared the relationships between forward and backward walking spatiotemporal measures to cognitive function (i.e., processing speed and verbal fluency), retrospective fall reports at six months, and balance. The critical finding of the current study was that backward walking measures, particularly in the dual-task condition, revealed greater decrements in walking performance compared to forward walking that better differentiate persons with MS from healthy controls. Additionally, backward walking measures were more strongly related to retrospective falls at six months whereas no forward walking measures were related. Though not adequately powered to comprehensively examine differences in walking performance among individuals with MS with higher (EDSS < 3) and lower (EDSS > 3) disability levels, a secondary exploratory analysis demonstrated that dual-task walking, in particular backward dual-task walking, better differentiated individuals with lower disability from healthy controls.

Backward walking velocity revealed significant differences between groups for both single-task (*p* = 0.015) and dual-task (*p* = 0.014) conditions. Interestingly, persons with MS had significant differences between single- and dual-task forward and backward walking velocities (*p* = 0.023; *p* = 0.004), whereas this difference was only apparent in the backward walking condition for healthy controls (*p* = 0.004; *p* = 0.827 for forward walking). Backward walking stride lengths were shorter for the MS group compared to controls in the forward walking dual-task condition and in the backward walking single-task condition. These findings are consistent with a similar study completed by Wajda and colleagues, in which motor differences were greater during backward walking and better distinguished individuals with MS from healthy controls than forward walking, and this effect was enhanced if individuals were administered a secondary cognitive task [30]. While the observed differences by Wajda were more robust in differentiating between individuals with MS and healthy controls, this study did not utilize matched control sampling. Importantly, we incorporated age- and sex-matched controls to better understand these differences, which may explain why our differences were not as robust across all dual-task conditions. Further, our findings are consistent with previous studies in MS that demonstrated impairments in dual-task walking performance were greater in persons with MS compared to healthy controls [12, 46].

Persons with MS demonstrated significant differences in double support time when shifting from single- to dual-task conditions in both forward (*p* = 0.001) and backward (*p* < 0.001) directions, whereas healthy controls displayed no differences. Importantly, this effect was heightened during backward walking, as persons with MS revealed significant differences in double support time when transitioning from forward to backward walking under both single- (*p* = 0.034) and dual-task (*p* < 0.001) conditions, whereas there were no differences in healthy controls (*p* = 0.440; *p* = 0.095). These findings are consistent with previous studies in MS that reported walking deficits and balance were more prominent in MS individuals during backward walking [29]. Additionally, backward walking is a nonautomatic motor skill that requires higher processing of both motor and cognitive resources [47]. The ability to complete complex motor tasks such as backward walking may be further hindered by cognitive-motor interference in persons with MS [10], and thus, it is not surprising that persons with MS exhibited increased double support time during backward walking whereas healthy controls did not.

Given the high demand of cognitive resources during dual-task walking and backward walking [47], our study examined the relationship between walking direction (single and dual task) and discrete domains of cognitive function, including information processing speed (SDMT) and verbal fluency (WLG). Interestingly, SDMT performance was only significantly associated with forward walking measures, including stride length in both single- (*r* = 0.505; *p* = 0.032) and dual-task conditions (*r* = 0.603; *p* = 0.008). WLG performance was not associated with any walking measures. These findings are consistent with previous studies that have shown relationships between forward walking performance and information processing speed [48]. However, our laboratory has previously shown that backward walking is related to SDMT (*r* = −0.61; *p* = 0.001) [32], and therefore, further research examining the relationship between backward walking and information processing speed with larger sample sizes is warranted. Additionally, unpublished data from our laboratory has shown relationships between backward walking performance and visuospatial memory on the Brief-Visuospatial Memory Test-Revised (BVMT-R), suggesting the involvement of different cognitive domains requiring greater study, as they may offer additional insight to effectively probing dual task and backward walking.

Backward walking measures were more strongly related to retrospective falls at six months whereas no forward walking measures were related. These findings reflect the sensitivity of backward walking and its potential to supplement the current clinical dual-task and fall risk assessments. Additionally, these findings agree with previous studies in the elderly [24, 25] and other neurodegenerative disease populations [26, 27] in which backward walking better identified fallers compared to forward walking. These findings also build upon our previous work in MS in which backward walking velocity exhibited the highest effect magnitude and specificity in differentiating fallers from nonfallers in individuals with MS [31].

We examined the relationship between forward and backward walking performance under single- and dual-task performance and balance, using the BBS. Interestingly, BBS demonstrated stronger relationships with forward walking measures than with backward walking measures. This is perhaps because the BBS comprises primarily measures of static and anticipatory balance control but does not require adaptive or reactive control. Future studies should explore whether balance tests incorporating adaptive or reactive control are more strongly related to measures of backward walking and backward dual-task walking.

An exploratory analysis indicated that individuals with lower disability (EDSS < 3) performed similar to healthy controls under single-task forward walking conditions, but that dual-tasks, specifically in the backward walking direction differentiates healthy controls from those with EDSS < 3 (Figure 2). These results build on previous findings in which forward walking dual-task assessment is better at differentiating between early diagnosed individuals with MS and healthy controls [46]. Therefore, it is critical for future studies to understand the abilities of dual-task assessment to detect early and subtle motor and cognitive symptoms in low-disability MS individuals to ensure early intervention with targeted rehabilitation.

Our findings are the first to elucidate that persons with MS exhibit greater deficits in backward walking single-task and dual-task walking performance compared to forward single-task and dual-task walking performance when compared to age- and gender-matched healthy controls. Additionally, these findings are the first to demonstrate the potential for backward walking dual-task assessment to sensitively detect fall risk in persons with MS. Importantly, the backward walking dual-task measures described in this work are clinically feasible, easy to administer, and could be immediately scalable for clinical use as a sensitive clinical outcome tool to use in addition to current methods to detect underlying impairments in key domains relevant to MS (i.e., cognitive function, fall risk, balance). However, a larger sample size with comprehensive multidomain cognitive testing, prospective falls monitoring, and dynamic balance assessment is needed to further elucidate clinical utility and validity for backward walking dual-task assessment in MS.

### 4.1. Limitations

Limitations of this study include its small sample size of 32 individuals (eighteen individuals with relapsing-remitting MS and fourteen age-matched healthy controls), which may not generalize to ambulatory persons with progressive subtype. However, these limited data satisfy prior gaps in knowledge regarding the relationship between backward walking dual-task assessment and falls, as well as successfully age- and sex-matching healthy controls while observing differences between forward and backward walking dual-task measures. This study relied on retrospectively collected data on falls at six months. Several studies in MS highlight the underestimation of falls with retrospective recall [21, 49], possibly due to high prevalence of cognitive dysfunction [50]. Therefore, it is critical that future studies consider the use of technology for prospective reporting of falls (i.e., smart phone applications, websites, wearable devices) to increase accuracy and predictive validity of fall data collection. Further, the best method of cognitive interference to detect impairment in dual-task assessment remains unidentified [14]. Thus, larger scale studies examining dual-task assessment are needed to validate the discrete measures used to generate cognitive interference.

This study was limited to two discrete measures of cognition, namely, the SDMT to measure information processing speed and the WLG to measure verbal fluency and semantic memory, and therefore did not evaluate other domains of cognition that are known to be impacted by MS (i.e., attention, visuospatial memory, executive function) and have been related to motor measures and fall frequency in MS [51]. Additionally, the domains of cognition suggested to be integrated with motor control (i.e., spatial navigation) should be incorporated into future studies. The level of education could also impact cognitive performance and should therefore be matched when recruiting control subjects in the future. This study was also limited to walking, static balance, and cognitive measures and thus, did not evaluate other factors that could heavily influence the impairments that were observed in persons with MS, including dynamic balance control, spasticity, and fatigue.

## 5. Conclusion

This study demonstrated that differences in MS and healthy controls are more pronounced during backward walking compared to forward walking. Importantly, we incorporated age- and sex-matched controls to better understand these differences. Future work with a larger sample size is needed to validate the clinical utility of backward walking and dual-task assessments and mitigate the limited sensitivity of the current dual-task assessments that primarily rely upon forward walking. Based on our data, larger scale studies could leverage identification of definitive variables that are easily measurable in the clinic setting (i.e., velocity) along with respective dual-task walking assessment cutoff scores for clinical use. Additionally, studies aimed at developing a comprehensive understanding of potential mechanisms (i.e., brain pathology and specific cognitive correlates) underlying the impairments observed in dual-task assessment and more specifically, backward walking dual-task assessment, would further enhance targeted rehabilitation interventions. Our findings suggest that backward walking and dual-task assessment may better differentiate persons with MS and healthy controls, providing additional tools to supplement the current standard of forward walking assessment and warrants further research.

## Figures and Tables

**Table 1:** Main effects and interactions for spatiotemporal measures of gait.

Main effects	Velocity (m/s)				Stride length (cm)				Double support time (s)			
	Mean (SD)	*F*	*p*	*η* ^2^	Mean (SD)	*F*	*p*	*η* ^2^	Mean (SD)	*F*	*p*	*η* ^2^
Group												
MS	**0.97 (0.40)**	**4.5**	**0.043**	**0.13**	**104.87 (26.26)**	**4.36**	**0.046**	**0.13**	0.50 (0.33)	3.5	0.071	0.12
Control	**1.17 (0.33)**				**116.96 (20.94)**				0.37 (0.12)			
Direction												
Forward	**1.28 (0.32)**	**255.6**	**<0.001**	**0.90**	**127.13 (16.86)**	**329.8**	**<0.001**	**0.92**	**0.36 (0.11)**	**10.5**	**0.003**	**0.27**
Backward	**0.84 (0.30)**				**93.01 (18.87)**				**0.53 (0.34)**			
Condition												
Single-task	**1.10 (0.39)**	**9.2**	**0.005**	**0.24**	109.43 (27.23)	2.1	0.160	0.07	**0.40 (0.22)**	**12.2**	**0.002**	**0.30**
Dual-task	**0.99 (0.39)**				107.54 (25.88)				**0.47 (0.31)**			
Group x direction												
MS forward	1.21 (0.33)	3.6	0.068	0.11	122.49 (17.61)	1.3	0.263	0.04	0.38 (0.12)	3.9	0.059	0.12
MS backward	0.72 (0.30)				86.76 (20.82)				0.63 (0.42)			
Control forward	1.37 (0.30)				133.09 (14.00)				0.34 (0.10)			
Control backward	0.98 (0.23)				100.82 (12.54)				0.40 (0.12)			
Group x condition												
MS single-task	1.04 (0.40)	0.87	0.359	0.03	106.09 (25.77)	0.31	0.584	0.01	0.45 (0.27)	1.7	0.199	0.06
MS dual-task	0.91 (0.39)				103.62 (27.06)				0.55 (0.38)			
Control single-task	1.21 (0.29)				117.64 (19.19)				0.34 (0.08)			
Control dual-task	1.14 (0.37)				116.28 (22.89)				0.39 (0.14)			
Direction x condition												
Forward single-task	1.33 (0.29)	1.2	0.311	0.06	128.11 (15.33)	0.94	0.341	0.03	**0.34 (0.09)**	**14.2**	**0.001**	**0.33**
Forward dual-task	1.23 (0.35)				126.15 (18.47)				**0.38 (0.13)**			
Backward single-task	0.90 (0.30)				94.18 (17.59)				**0.47 (0.28)**			
Backward dual-task	0.78 (0.28)				91.80 (20.32)				**0.58 (0.39)**			

Bolded values represent significant effects.

## Data Availability

Readers may contact the authors for access to the deidentified dataset.

## Additional Points

The OSF Registration is dated 7/13/19 and is available for review at: https://osf.io/6j5qt/?view_only=6768b579945c469cb710cba8068a4343

## Supplementary Materials

Supplementary Materials STROBE Statement—Checklist of items that should be included in reports of cross-sectional studies.
